# Survey of northern informal and formal mental health practitioners

**DOI:** 10.3402/ijch.v72i0.20962

**Published:** 2013-08-05

**Authors:** Linda O'Neill, Serena George, Stefanie Sebok

**Affiliations:** 1 Counselling Program, School of Education, University of Northern British Columbia, Prince George, BC, Canada; 2 Queen's University, Kingston, ON, Canada

**Keywords:** northern, mental health, informal and formal practitioners, research

## Abstract

**Background:**

This survey is part of a multi-year research study on informal and formal mental health support in northern Canada involving the use of qualitative and quantitative data collection and analysis methods in an effort to better understand mental health in a northern context.

**Objective:**

The main objective of the 3-year study was to document the situation of formal and informal helpers in providing mental health support in isolated northern communities in northern British Columbia, northern Alberta, Yukon, Northwest Territories and Nunavut. The intent of developing a survey was to include more participants in the research and access those working in small communities who would be concerned regarding confidentiality and anonymity due to their high profile within smaller populations.

**Design:**

Based on the in-depth interviews from the qualitative phase of the project, the research team developed a survey that reflected the main themes found in the initial qualitative analysis. The on-line survey consisted of 26 questions, looking at basic demographic information and presenting lists of possible challenges, supports and client mental health issues for participants to prioritise.

**Results:**

Thirty-two participants identified various challenges, supports and client issues relevant to their mental health support work. A vast majority of the respondents felt prepared for northern practice and had some level of formal education. Supports for longevity included team collaboration, knowledgeable supervisors, managers, leaders and more opportunities for formal education, specific training and continuity of care to support clients.

**Conclusion:**

For northern-based research in small communities, the development of a survey allowed more participants to join the larger study in a way that protected their identity and confidentiality. The results from the survey emphasise the need for team collaboration, interdisciplinary practice and working with community strengths as a way to sustain mental health support workers in the North.

Research and information on northern mental health practice in Canada is limited, and what is available comes from the professions of social work and nursing. The challenges faced in the delivery of mental health and wellness support in the North are better understood through this profession-specific research (1). Yet the situation of both formal and informal practitioners who provide mental health support throughout northern Canada is scarce. Lay counsellors, drug and alcohol counsellors, community counsellors, child and youth care workers, elder counsellors, social workers, nurses and other community helpers, both Aboriginal and non-Aboriginal, provide essential psychological and emotional support to clients or family members who live in the North. Previous experience in the North led the research team to use a broad lens and consider the situation of practitioners from many different professions and paraprofessionals from all walks of life in their work of supporting northerners experiencing a broad range of mental health issues.

The existing research often focuses on specific professions, such as social work and nursing, who provide various levels of mental health support in northern communities (2–4). The research team decided to approach mental health support in the North using a more general, inclusive model for recruitment, understanding that much of the supportive work is done by professionals in many helping professions and by informal helpers and paraprofessionals. Informal and formal helping practitioners play a key role in supporting the well-being of community members, and in answering the call to hear more voices from helpers in the field (5), this research focused on the localised knowledge of mental health providers throughout the North. The main objective of the 3-year study was to document the situation of formal and informal helpers in providing mental health support in isolated northern communities in northern British Columbia (BC), northern Alberta, Yukon, Northwest Territories (NWT) and Nunavut. In order to better understand mental health and wellness support in the North, the following question informed both the qualitative and quantitative phases of the research: What are the life-career issues, supports, challenges and barriers for formal and informal helping practitioners in northern communities?

In northern communities, lack of anonymity and personal privacy is a major challenge. Fundamental issues for isolated practitioners include high visibility with a loss of privacy and anonymity when living and working in small isolated communities (6, 7). Northern communities are often locations where people know everyone else in the community whether they want to or not (8). Professionals who come to northern communities often feel as though they are constantly observed and often critically viewed by some community members (2). Separating the practitioner's personal and professional life and one's membership in a health system from one's presence as a new community member in a northern community is extremely difficult (9). In many small northern communities, helping practitioners may be related to other community members. The challenge of confidentiality in northern practice hinges on the difficulty of ensuring client privacy since most community members know each other (10) and informal conversations abound, adding to what Schank and Skovholt (11) refer to as the small world hazards found in helping professions in small communities. The potential of increased isolation of community practitioners is due to their concerns about sharing confidential information. The problems of helping practice in small communities mirror related problems in northern research.

## Material and methods

### Background

The multi-method approach used in this multi-year research study involved the use of qualitative and quantitative data collection and analysis methods. Based on previous research (12), the need to protect the anonymity and confidentiality of practitioner participants was foremost in the research development process. Ethical research practice in small northern communities needs to insure participant confidentiality, suggesting acute sensitivity in the writing of the qualitative analysis piece and the need for the development and use of a survey. The intent of developing a survey based on the in-depth interviews was to include more participants in the research who would be difficult to access for face-to-face interviews due to remote locations, and those working in small communities who would be concerned regarding confidentiality and anonymity due to their high profile within smaller populations.

This high profile situation of many helping professionals in small communities forces researchers to take extra steps to ensure confidentiality and anonymity through interviewing participants in communities other than their own, not identifying any communities or specific agencies and by using anonymous survey methods of data collection. We are presenting limited, broad demographics due to our concerns regarding the small population of northern communities and the resultant common problems around assuring confidentiality, especially to those people who provide mental health and wellness services and who may be the only person in the community doing this work.

## Research ethics

The study received Research Ethics Approval from the University of Northern British Columbia, the University of Lethbridge, a Scientific License from the Yukon Government Department of Tourism and Culture and a Research Licence from the Aurora Research Institute, Aurora College, NWT.

## Survey development and analysis

Based on the in-depth interviews from the qualitative phase of the project, the research team developed a survey that reflected the main themes found in the initial qualitative analysis completed after each interview by research assistants during 2010 and 2011 (Appendix A). The initial thematic analysis was the framework for the team to develop the key questions on the survey, with the analysis reflecting the main points and concerns from the 20 participants who took part in the in-depth interviews during the qualitative phase of the project (13). In using an initial analysis of qualitative interviews to develop the survey, the intent was to avoid any assumption on the part of the research team that we knew the most relevant topics and appropriate questions that would allow for a greater understanding of the practitioners who provide mental health support throughout the North. Rather, the completed survey presents questions grounded in northern practitioner experience. This on-line survey developed through Fluid Surveys Canada consisted of 26 questions, some looking at basic demographic information and others presenting lists of possible challenges and supports for participants to prioritise. At the end of the survey, a textbox was included for respondents to provide additional information that was relevant, but not necessarily captured in the designed survey questions.

All of the data gathered from the on-line survey were exported and descriptive statistics were calculated using Microsoft Excel. The survey results provided demographic information and descriptive statistics that were analysed in relation to the original qualitative data.

## Results

Thirty-two northern practitioners, both informal and formal mental health providers, living in northern BC, northern Alberta, Yukon, NWT and Nunavut completed the on-line survey. The sample consisted of 23 females and 9 males, ranging in age from 25 to 58 years of age, with the highest participation from the NWT. Only 2 of the survey participants identified as being Aboriginal (6.25%). This number has significant implications for this study and general practice in the North that will be elaborated on in the conclusion.

This sample of mental health supporters surveyed revealed that the average number of communities served by an individual supporter was 2.75, with the largest number of communities served by 1 person being 8. Furthermore, 34% of respondents are presently working on a contract basis. Although some of the practitioners travel to communities, the majority of the respondents live in the community where they work. The range of experience among practitioners varied from less than 1 year to almost 40 years; however, most people reported having 10 years of experience working in the North. One of the most surprising findings was that regardless of the amount of experience participants had, 81% of the practitioners felt prepared for work in northern communities, with this finding directly contradicting some existing research. Table I expands upon this result by illustrating the frequency of factors that contribute to the work of individuals in the North.

**Table I:** Factors contributing to individuals’ mental health work in the north

Questions	First response	Second response	Third response
What training do you have?	Formal education	Work experience	Life experience
What attributes make you effective?	Communication	Empathy	Flexibility and personal awareness
In what ways do you feel supported?	Financially	Socially	Mentally and emotionally
What resources are available to you?	Work space	Training	Case consultation
Who do you collaborate with?	Colleagues	Health care providers	Social services

Knowledge and experience both factored into practitioners view of training. The number of respondents having some level of formal education (91%) may reflect the majority feeling of preparedness for northern mental health work. Other aspects such as work experience, life experience, volunteer work, and mentoring were also listed as essential elements of practitioners training. Practitioners listed communication skills as being the most important personal attribute for northern practice closely followed by empathy, then flexibility and personal awareness. The importance of communication skills rather than specific therapeutic skills echoes some of the discussion from the qualitative interviews where practitioners described themselves as over trained and having to step back to meet clients where they were currently at a rapport-building stage. The primary way that individuals working in the North feel supported is financially; however, only about half of the respondents felt supported in other ways (i.e. socially, mentally, emotionally, physically, spiritually and culturally). Practitioners identified resources available to them at this time as workspace, training and workshops, and case consultation. Other key resources such as community support, cultural guidance and language interpreters were only reported by the participants approximately 20% of the time. When asked about what resources are essential for mental health supporters working in the North, 75% of respondents listed training and workshops followed by 50% listing mentoring and supervision. Finally, a key area of concern in northern practice is collaboration. The respondents indicated that their main source of collaboration was with their colleagues, health care providers and social services 80–90% of the time.

Working in the North presents barriers and challenges to mental health support as previously noted. According to the survey respondents, challenges impacting their work included workload and complexity of client issues, geographical and social isolation, and high personnel and staff turnover (53%). The most significant barriers for practitioners in their work included geographical and social isolation and workload, mirroring previous research. Other barriers identified included lack of self-care resources, lack of sustained programme funding and complexity of client issues. In looking at sustained practice and longevity, respondents identified many options for more effective practice, including developing collaborative relationships, working with community strengths, and awareness of both the challenges and the supports available to them in the communities. Supports for longevity agreed upon by respondents included team collaboration, knowledgeable supervisors, managers, leaders, and more opportunities for formal education, specific training and continuity of care to support clients.

Table II outlines the type and frequency of issues that mental health supporters provide care for. The following issues listed in Table II highlight major client issues as reported by respondents of the survey.

**Table II:** Frequency of major client issues as reported by mental health supporters

Issues	Frequency (%)
Schizophrenia/Psychosis	28
Disabilities (Physical and Cognitive including FASD)	41
Personality Disorders/Mood Disorders	53
Sexual Assault	56
Psycho-Educational	59
Anxiety Disorders	63
Child Abuse	66
Self-Harming Behaviors (Cutting, Burning, etc.)	66
Parenting Issues	69
Depression	72
Suicide	72
Trauma (PTSD, Historical, Intergenerational, Complex)	72
Addictions	78
Crisis Intervention	78
Grief and Loss	78

In the face of complicated client issues, practitioners identified codes of ethics, lived experience and standards of practice as being used as their guide in ethical decision making. Despite the strong use of codes of ethics by the practitioners, the respondents suggested that codes related to dual relationships, practitioner competence and confidentiality needed to be changed to reflect northern practice. The practitioners also identified family counselling services, trauma awareness, emphasis on healthy living and mentoring programmes as being the best support services for their clients. The need for family counselling indicated a major lack of such services currently in the North. When asked what provides the motivation for practitioners to continue to provide mental health support in the north, 82% of the practitioners listed commitment to clients as being the main motivator to keep doing the work. Other motivators such as hope, commitment to community, and personal fulfilment were also mentioned by the participants.

At the end of the survey, a textbox was included for respondents to provide additional information. In these sections, participants discussed the need for more support and training for Aboriginal paraprofessionals that would be culturally relevant. In line with personal motivation, 1 participant reflected that practitioners really need to understand why they are doing the work they do, echoin a theme from the qualitative analysis. Another participant stated that working to shift the doubt and suspicion related to making change took the most energy in practice. The essential need for forming collaborative partnerships where the diverse aspects of helping in northern communities are valued was highlighted by several respondents. The majority of these responses emphasised the importance of interdisciplinary work in northern communities and the differences between providing mental health and wellness support in the North versus providing similar services in more populated southern regions of Canada.

## Conclusion

Through the safety of an anonymous survey method of data collection, participants were supported in contributing to the larger research project. The survey findings added specific information to the guiding question on the life-career issues, supports, challenges and barriers for formal and informal helping practitioners in northern communities. Information on the complexity of client issues and what supports are currently in place and those that are needed in small communities adds to an improved understanding of what mental health support looks like in the North.

Despite the high Aboriginal population throughout the North, the low number of Aboriginal respondents may indicate that this survey tapped into a majority of professionals who at one time came into northern communities from “outside”. This result may also indicate hiring criteria for professional mental health positions, reflecting the lack of post-secondary opportunities for northerners living in remote communities. Participants requested more culturally relevant training for Aboriginal support workers, suggesting that there is work to do in all educational endeavours on mental health. The research team will continue to refine research strategies to reach more informal mental health supporters, particularly those of Aboriginal descent.

The findings from the survey again emphasise the need for collaboration, supervision, and mentorship in order to better support practitioners and sustain mental health work in the North. The next step will be to find ways to network all people providing mental health support in the North to alleviate the inherent isolation of such work in a northern context.

## Appendix A

Northern Mental Health and Wellness Survey

The first part of this survey provides some general information about you.

1. What is your gender?

  Male

  Female

  Other

2. How old are you?

3. What territory or province do you work in?

  Yukon

  NWT

  Nunavut

  B.C.

4. What type of applicable education do you have?

  Formal education (degree, diploma, certified course)

  Work experience

  Life experience

5. How many years have you provided mental health support in the North?

6. How many communities do you provide services to?

7. Do you work on a contract basis?

  Yes

  No

8. What personal attributes make you effective in your role as a helper?

  Flexibility

  Creativity

  Personal awareness

  Empathy

  Communication skills

  Cultural sensitivity

  Resiliency

  Humour

9. What prepared you for this work?

  Formal education

  Mentoring

  Volunteer work

  Life experience

  None of the above*

10. Indicate which of the following supports you collaborate with in your work.

  Colleagues

  Health care providers

  Social services

  Law enforcement

  School personnel

  Elders

  Other

11. Do you live in a community where you provide mental health support?

  Yes

  No

  *Branching, if Q8 answer was no*

12. Is adequate housing available to you a community where you live and work?

  Yes

  No

13. Given the services you provide, please indicate the ways in which you as a practitioner feel supported.

  Financially

  Physically

  Socially

  Culturally

  Mentally

  Emotionally

  Spiritually

14. Please indicate the resources that are available to you in your work as practitioner.

  Training and workshops

  Financial resources

  Health services

  Work space

  Case consultation

  Mentoring/supervision

  Community support

  Accessible cultural guidance

  Peer/family support

  Language interpreters*

15. What are the 3 most important resources necessary for you in this line of work?

16. In what ways are you involved in your community?

  Volunteer

  Sports

  Arts

  Education

  Committee work

  Clubs and organisations

**This part of the survey asks about what you see as being barriers and supports for formal mental health practitioner**.

17. Please indicate which of the following challenges are most applicable to your work.

  Insufficient training opportunities

  Racism

  Isolation (geographical or social)

  Lack of sustained programme funding

  Minimal cultural understanding

  Heavy workload

  Complexity of client issues

  Limited professional collaboration

  Poor driving conditions

  Not enough self-care resources

  Unmet personal expectations

  High personnel/staff turnover

  Language barrier

  Others, please specify

18. What are the 3 most challenging barriers you face in your work?

19. Which of the following options do you see as practical for making working in the North more effective?

  Developing collaborative relationships

  Working with community strengths

  Living in the community that you work in

  Accessing external resources (colleagues, friends, professional consultation, etc.)

  Participating in traditional or non-traditional community practices

  Tailoring your practice to the needs of the community

  Awareness of challenges and supports available

  Increased aboriginal cultural understanding

  Other

20. What Supports would help you do the work you do longer?

  More opportunities for formal education/specific training

  Long-term job security

  Relief time

  Increased financial support

  Knowledgeable supervisor/manager/leader

  Validation of the services you provide

  Team collaboration

  Continuity of care for supporting clients

  Other

21. Please indicate which of the following issues you provide client support for.

  Trauma (PTSD, historical, inter-generational, complex)

  Grief and loss

  Addictions

  Depression

  Anxiety disorders

  Self-harming behaviors (cutting, burning, etc.)

  Suicide

  Sexual assault

  Personality disorders/mood disorders

  Schizophrenia/psychosis

  Parenting issues

  Child abuse

  Disabilities (physical, cognitive, including FASD)

  Psycho-education

  Crisis intervention

22. Please indicate what support services you feel would best help the clients you work with.

  Trauma awareness

  Follow-up support

  Community activities (community centre)

  Emphasise healthy living

  Family counselling services

  Mentoring Programmes

  Specific skill development

  Parent support

  Alternative education options

**This part of the survey asks about the motivation and ethics surrounding your work as formal mental health practitioner**.

23. What guides your decision making as a formal mental health practitioner?

  Professional codes of ethics

  Standards of practice

  Agency rules and regulations

  Cultural beliefs and guidelines

  Spirituality or religion

  Moral beliefs and values

  Lived experience

24. What changes would you make to your professional code of ethics that would make it relevant to Northern practice?

  Confidentiality

  Practitioner competence

  Client's rights

  Supervision

  Dual relationships/boundaries

  Informed consent

  Bartering for services

  Other

25. Please indicate what motivates you to continue to do this work in the North?

  Sense of duty

  Commitment to clients

  Financial reward

  Advocacy

  Commitment to the community

  Community/leadership request

  Autonomy

  Personal fulfilment

  Hope

  Other

26. Please include any other information that you believe is important to Northern mental health/wellness support.
